# Mechanism of Ferroptosis and Its Relationships with Other Types of Programmed Cell Death: Insights for Potential Therapeutic Benefits in Traumatic Brain Injury

**DOI:** 10.1155/2022/1274550

**Published:** 2022-08-24

**Authors:** Qiuyu Pang, Lexin Zheng, Zhiyang Ren, Heng Xu, Hanmu Guo, Wenqi Shan, Rong Liu, Zhiya Gu, Tao Wang

**Affiliations:** Department of Forensic Science, Suzhou Medicine College of Soochow University, Suzhou 215123, China

## Abstract

Traumatic brain injury (TBI) is a serious health issue with a high incidence, high morbidity, and high mortality that poses a large burden on society. Further understanding of the pathophysiology and cell death models induced by TBI may support targeted therapies for TBI patients. Ferroptosis, a model of programmed cell death first defined in 2012, is characterized by iron dyshomeostasis, lipid peroxidation, and glutathione (GSH) depletion. Ferroptosis is distinct from apoptosis, autophagy, pyroptosis, and necroptosis and has been shown to play a role in secondary brain injury and worsen long-term outcomes after TBI. This review systematically describes (1) the regulatory pathways of ferroptosis after TBI, (2) the neurobiological links between ferroptosis and other cell death models, and (3) potential therapies targeting ferroptosis for TBI patients.

## 1. Introduction

Traumatic brain injury (TBI), which is usually caused by a violent event that disrupts normal brain structure and/or function, is a serious public health problem with a high incidence, high morbidity, high mortality, and high economic burden [1]. Each year, around 50 million people worldwide experience TBI, and approximately 50% of the population will experience one or more TBI in their lifetime [2, 3]. In China, the incidence of TBI is 55.4–64.1 cases per 100,000 people each year, which is equivalent to about 770,060–890,990 new cases of TBI annually [2, 3]. It is estimated that TBI-related deaths account for about 30–40% of all injury-related deaths and that TBI will become the fourth leading cause of disability-adjusted years in 2030 [4]. TBI is a chronic condition with serious long-term consequences including cognitive deficits, neurodegenerative diseases, epilepsy, and stroke [5]. An estimated 317 million people suffer from permanent sequelae of TBI in the United States, and the international treatment cost for TBI is about 400 billion USD annually, which is equal to 0.5% of the global annual GDP [2]. Although recent studies have identified therapies and drugs that can improve the outcomes of TBI in animal models, there has not yet been a clinical trial in humans due to the complex pathogenesis of TBI [6, 7]. As a result, identifying effective therapeutic targets and drugs for TBI is a focus of neuroscience research.

TBI is categorized into two types: primary brain injury and secondary brain injury. Primary brain injury refers to direct damage to the brain parenchyma during the initial impact that results in brain structural abnormalities, cerebrovascular dysfunction, and brain tissue necrosis; secondary brain injury refers to brain lesions that occur after the initial injury, which are mainly cerebral edema and cerebral hemorrhage [4, 6]. Compared with primary brain injury, secondary brain injury after TBI generally has more serious effects on patients. The pathophysiological mechanisms of secondary brain injury, including oxidative stress, endoplasmic reticulum stress (ERS), caspase activation, neuroinflammation, and amino acid metabolism disorder, are the main causes of neuronal cell death and cognitive dysfunction in TBI patients [8]. Previous studies have described that neuronal cell death after TBI may occur by apoptosis, necroptosis, autophagy, and pyroptosis [9]. However, recent research has reported that ferroptosis is also involved in secondary neuronal cell death and neurological dysfunction after TBI [10].

Ferroptosis, which refers to iron-dependent cell death induced by small molecules, is characterized by excessive accumulation of intracellular reactive oxygen species (ROS) that cannot be inhibited by inhibitors of apoptosis, pyroptosis, necroptosis, or autophagy, only by antioxidants and iron chelators [10, 11]. Ferroptosis is morphologically distinct from apoptosis, pyroptosis, autophagy, and necroptosis in that it is mainly manifested by decreased mitochondrial volume, increased bilayer membrane density, and a decrease or disappearance of the matrix, but an intact cell membrane and normal nucleus [10, 11]. In the pathophysiological process of TBI, the hemoglobin/heme in the hematoma releases iron that can trigger the generation of ROS via the Fenton reaction and attack mitochondrial inner membranes, leading to energy deficiency and cellular dysfunction [12, 13]. Xie et al. [14] observed the typical characteristics of ferroptosis, such as mitochondrial pyknosis and volume reduction, in injured cortex three days after experimentally-induced TBI using scanning electron microscopy. In recent decades, evidence of iron deposition has been reported in experimental TBI studies [14]. Recent animal experiments have also shown that inhibition of ferroptosis can effectively prevent neurodegeneration and neurological deficits after TBI, further suggesting that ferroptosis is involved in the pathophysiological process of TBI [15]. Given these findings, increasing research has been devoted to targeting ferroptosis to identify effective drugs and therapeutic targets for TBI.

## 2. The Ferroptosis Regulatory Pathway and Ferroptosis Activation after TBI

**Figure 1:**
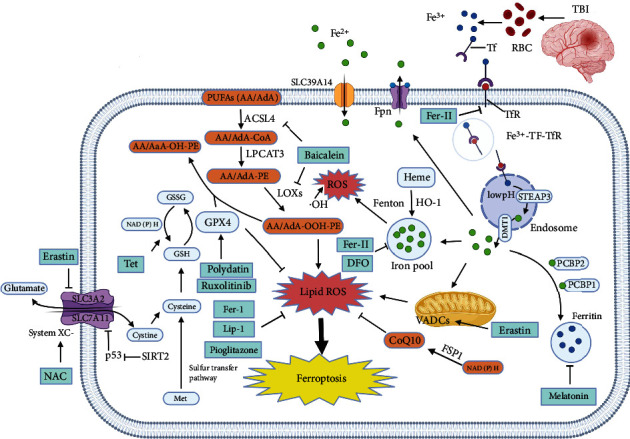
Diagram of ferroptosis pathway and its therapeutic target after TBI. Top right shows the iron metabolism pathway of ferroptosis. Extracellular Fe^3+^ is combined with Tf and the Fe^3+^-TF-TfR complex enters cell by endocytosis. In endosome, Fe^3+^ is reduced to Fe^2+^ at low pH. Fe^2+^ in the cytoplasm may be exported to the extracellular by Fpn, stored in Ferritin, and transported to mitochondria and other organelles to maintain their physiological functions. When Fe^2+^ overloads after TBI, a large of ROS are generated through Fenton reaction. Bottom left shows the system XC-/GSH/GPX4 pathway of ferroptosis. System XC- transports cystine into cell and glutamate out of cell by a one-to-one way. Cystine is reduced to cysteine and then transformed into GSH. GSH is required for GPX4 activity and reverses lipid peroxidation. TBI leads to GSH depletion and decreases GPX4 activity, resulting in more lipid peroxides. Top left shows lipid ROS pathway of ferroptosis. PUFAs such as AA and AdA in cytomembrane are vulnerable to oxidizing substances after TBI. PUFAs can transform into PEox under the catalysis of ACSL4, LPCAT3, LOXs, and other biological enzymes, resulting in lipid ROS and ferroptosis. In addition, other pathways such as p53, CoQ10, and VADCs are also involved in regulating ferroptosis. The drugs in the wathet blue box are ferroptosis regulators after TBI.

Iron dyshomeostasis, lipid peroxidation, and glutathione (GSH) depletion are considered as three important pathways of ferroptosis regulated by complex signaling pathways [16]. Lipid peroxidation and GSH deficiency have been widely demonstrated in both TBI animal models and patients [16]. Based on the findings that abnormal iron homeostasis can lead to a pathological state of the central nervous system (CNS) and abnormally elevated iron in the brain tissue of mild TBI patients, abnormal iron homeostasis may play an important role in TBI [17].  Figure 1 presents a summary of the regulatory pathways of ferroptosis reported in previous studies with the aim of better revealing the mechanism of ferroptosis after TBI.

### 2.1. Iron Metabolism Pathway

Extracellular iron, which is stored in transferrin (Tf) as Fe^3+^, binds to transferrin receptors (TfR) on the cell membrane to form the Fe^3+^-Tf-TfR complex, which is then endocytosed to form an endosome under physiological conditions [18]. The Fe^3+^-Tf-TfR complex is disrupted at a low pH in endosomes and releases Tf, TfR, and Fe^3+^; of these, Tf and TfR are recycled to the cell membrane, while Fe^3+^ is reduced to Fe^2+^ by action of the six-transmembrane epithelial antigen of prostate 3 (STEAP3). Alternatively, extracellular Fe^2+^ may be directly transported into the cytoplasm by SLC39A14. Recent studies have shown that Fe^2+^ in the cytoplasmic iron pool is regulated through the following steps to maintain iron metabolism [19]: (1) ferroportin (Fpn) mediates intracellular iron transport to extracellular space; (2) Fe^2+^ is transported and converted into Fe^3+^ by poly C-binding protein 1/2 (PCBP1/2) and stored in ferritin, the function of which depends on the complex of 24-heteromultimers of light (FTL) and heavy (FTH) chains; (3) Fe^2+^ is transported to organelles like mitochondria to maintain its physiological function; and (4) Fe^2+^ is released in the cytoplasm through heme via heme oxygenase-1 (HO-1) [20]. When Fe^2+^ is overloaded in the iron pool, the iron metabolism balance is broken and the excessive “free iron” generates a large amount of ROS via the Fenton reaction [21, 22], which can lead to cell damage and even death due to disruption of intracellular lipids, proteins, and nucleic acids. The above process is termed ferroptosis.

In a previous study from our group [23], iron deposition was observed to increase 3–7 days after TBI in a mouse model. We [24] also found that iron deposition in the injured cortex was significantly higher than that in control cortex 21 days after TBI. Furthermore, we demonstrated that the iron metabolism pathway-related proteins TfR1, FPN, FTH, and FTL were temporally expressed in injured cortex, with TfR1 and FPN expression peaking 6–12 h after TBI and FTH and FTL expression peaking 3–7 days after TBI [23, 24]. These findings suggest that the amount of iron deposition and expression of iron metabolism pathway-related proteins in the injured area correlate with the time since TBI. We [23] also discovered that specific knockout of *Fth* in neurons resulted in more obvious iron deposition, more severe neuronal degeneration, and higher levels of toxic substances such as 4-hydroxynonenal (4-HNE) in the injured cortex post-injury compared with the Fth-floxed TBI group. Zhang et al. [25] showed that the iron content and levels of FTL, FTH, and Tf in the brain tissue of mice were significantly increased even on the 28th day after TBI. Taken together, these findings imply that the iron metabolism balance is disrupted regardless of in the acute or chronic phase after TBI and results in iron deposition, subsequently leading to lipid peroxidation and ferroptosis. In addition, elevated serum ferritin levels were found to be negatively correlated with serial Glasgow Coma Scale (GCS) scores in severe TBI patients, but positively correlated with increased mortality of TBI patients in intensive care unit (ICU) [26, 27].

### 2.2. Lipid ROS Pathway

Lipid peroxidation refers to the process in which oxygen or hydrogen peroxide molecules provide hydrogen peroxide groups and then inserts hydrogen peroxide groups into lipid molecules. This process is closely related to a cell's sensitivity to ferroptosis. Polyunsaturated fatty acids (PUFAs) are essential components of cell membrane phospholipids and are involved in the regulation of multiple biological functions including inflammation, immunity, synaptic plasticity, and cell growth [28, 29]. In the process of ferroptosis, PUFAs—especially arachidonic acid (AA) and adrenic acid (AdA)—contain easily extractable diallyl hydrogen atoms that are highly prone to peroxidation, leading to destruction of the lipid bilayer and affecting membrane function. The free-PUFAs are esterified into membrane phospholipids which then combine with CoA and are converted into AA/AdA-CoA under the action of acyl-CoA synthetase long-chain family member 4 (ACSL4) and form AA/AdA-phosphatidylethanolamine (AA/AdA-PE) under the catalysis of lysophosphatidylcholine acyl transferase 3 (LPCAT3) [30]. AA/AdA-PE can be synthesized into lipid peroxide, AA/AdA-hydroperoxide-PE (AA/AdA-OOH-PE), via the enzymatic pathway involving lipoxygenase (LOX) and the nonenzymatic pathway called the Fenton reaction. Lipid peroxides are then decomposed into malondialdehyde (MDA) and 4-HNE under the action of cyclooxygenase-2 (COX2), nicotinamide adenine dinucleotide phosphate oxidases 2 (NOX2), etc., which affects the fluidity and permeability of the cell membrane [28, 29, 31]. Thus, PUFAs, ACSL4, LPCAT3, LOX, Fenton reaction, and ROS are all involved in the above process, which ultimately promotes ferroptosis.

Since the CNS is rich in lipids, it is prone to lipid peroxidation damage. Previous studies have shown that lipid peroxidation is involved in TBI and correlates with injury severity and mortality [32]. High expression of free-PUFAs in the serum of TBI mice was found to further activate lipid peroxidation and make damaged brain tissue more prone to ferroptosis [33]. Our previous studies [23] revealed that COX2, NOX2, and 4-HNE show temporal expression in the damaged cortex, with COX2 and NOX2 expression peaking 1 day after injury and 4-HNE expression peaking 7 days after injury. Xiao et al. [34] reported significantly increased levels of MDA and Fe^2+^ in the injured cortex, as well as increased expression of the ferroptosis-related genes *Acsl4* and *Nox2* in the acute phase of TBI. The above results indicate that lipid peroxidation-mediated ferroptosis is activated after TBI.

### 2.3. The System XC-/GSH/GPX4 Pathway

Dixon et al. [10] established an organotypic hippocampal slice culture model and used glutamate to induce excitotoxic cell death. They found that this process was oxidative and iron-dependent and thus inferred that glutamate-induced cell death and ferroptosis may share some common signaling pathways. Glutamate-induced cell death can be initiated through two pathways, namely, the influx of calcium ions and the inhibition of the cystine that depends on the cystine glutamate transporter receptor known as system XC- [35]. In addition, an experiment by Geng et al. demonstrated that the ferroptosis inducer erastin could inhibit system XC- [28]. The above studies prove system XC- plays an important role in ferroptosis.

System XC- is composed of light chain xCT (encoded by *SLC7A11*) and heavy chain 4F2 (encoded by *SLC3A2*) [36] and exchanges extracellular cystine for intracellular glutamate or cysteinate at a ratio of 1 : 1 [37]. GSH, an essential intracellular antioxidant, is formed by glutamate, cysteine, and glycine under *γ*-glutamate-cysteine synthetase (*γ*-GCS) and glutathione synthetase (GS) [28]. Glutathione peroxidase 4 (GPX4), which is a kind of selenoprotein that contains one selenocysteine at the active site and seven cysteines, is considered as the only GPX capable of eliminating biofilm lipid peroxidation and has a unique ability to inhibit ferroptosis [38]. GSH is a necessary cofactor for GPX4 to eliminate lipid peroxidation. Under the action of GPX4 (a GSH-dependent enzyme downstream of system XC-), GSH is converted to oxidized glutathione (GSSG), while lipid peroxide (L-OOH) is reduced to the corresponding alcohol (L-OH); this inhibits the production of lipid ROS, which helps maintain the fluidity of the plasma membrane so as to protect cells from damage or even death caused by lipid peroxide [28]. Inhibition of system XC- ultimately leads to GSH depletion because SLC7A11 is overexpressed. This affects cystine uptake and reduces cysteinate, the rate-limiting substrate for GSH synthesis, which is reduced by intracellular cystine under GSH or thioredoxin reductase 1 (TrxR1) [39]. GPX4, which is regulated by GSH and mevalonate (MVA) pathway by regulating selenocysteine tRNA maturation [40], can reduce L-OOH to related alcohol L-OH, which also inhibits the generation of lipid ROS to maintain plasma membrane fluidity and protect cells from lipid peroxide-induced damage and death.

Given the findings that system XC- is regulated by glutamate and that the extracellular glutamate concentration was significantly increased and system XC- was inhibited after TBI [41], as well as the important role of system XC- in ferroptosis and its close relationship with GSH, numerous studies have investigated the system XC-/GSH/GPX4 pathway as a way of preventing ferroptosis after TBI. We [23] observed that xCT protein was temporally expressed in the injured cortex, peaking 1 day after injury in a mouse TBI model. Our prior study [24] found that GPX4 protein significantly decreased in the acute phase after TBI, returning to the normal level 7 days after injury. An experimental study also showed that GSH decreased or was even depleted after TBI [23]. Consistent with this above finding, decreased GSH has been observed in the serum of patients with clinically mild TBI and is correlated with posttraumatic epilepsy [42]. Choi et al. [43] reported that GSH intake decreased and neuronal cell death increased significantly in a mouse TBI model after knocking out the *excitatory amino acid carrier type 1* gene (*EAAC1*), which encodes a high affinity glutamate transporter. The above studies show that the system XC-/GSH/GPX4 pathway plays an important role in the occurrence of ferroptosis after TBI and suggest that acting on the above targets may improve the prognosis of TBI patients.

### 2.4. Other Regulatory Pathways

The tumor suppressor p53 plays a vital role in the occurrence and development of tumors by mediating cell cycle inhibition, senescence, and apoptosis and is also related to ferroptosis [44]. P53 reduces cystine intake and GSH synthesis, inhibits GPX4 activity, weakens cell antioxidant capacity, increases lipid ROS, and causes ferroptosis by inhibiting expression of the system XC- subunit SLC7A11 [44]. Notably, variants in the *TP53* gene (encoding p53) appear to predict prognosis in patients with severe TBI [45]. Sirtuins are a nicotinamide adenine dinucleotide (NAD^+^) dependent and evolutionarily conserved family of deacetylases. Previous studies have shown that sirtuin 2 (SIRT2) has a neuroprotective effect against TBI [46]. Gao et al. [47] found that SIRT2 inhibition can upregulate p53 expression and acetylation in a mouse TBI model and aggravate ferroptosis. Interestingly, *p53* knockout can rescue SIRT2 inhibition-induced ferroptosis, suggesting that SIRT2 can inhibit p53-mediated ferroptosis and exert neuroprotective effects. The *GLS2* gene is a transcription target of p53 that can promote p53-dependent ferroptosis; however, in some cases, *p53* inhibits ferroptosis by blocking dipeptidyl-peptidase 4 (DPP4) activity in a transcription-independent manner [48].

Ferroptosis-suppressor-protein 1 (FSP1) is a GSH-independent coenzyme Q10 (CoQ10) plasma membrane oxidoreductase containing an N-myristoylation signal and a flavoprotein oxidoreductase domain, which are both necessary for its function in suppressing ferroptosis [49]. The FSP1/CoQ10/NAD(P)H pathway thus acts as an independent system that inhibits phospholipid peroxidation and ferroptosis together with GPX4 and GSH [49]. Reducing CoQ10, which is a fat-soluble antioxidant formed by the conversion of oxidized CoQ10 catalyzed by FSP1, can inhibit peroxidation and ferroptosis, thereby preventing oxidative damage to protein, lipids, and DNA [50]. It has been shown that FSP1 can utilize NAD(P)H to regenerate CoQ10 and inhibit ferroptosis independently from GSH [50]. At present, FSP1 is the only known inhibitor of ferroptosis that regulates GPX4 deficiency, whereas GPX4 provides compensatory inhibition in *FSP1* gene knockout mice [51]. However, the involvement of FSP1 in the regulation of ferroptosis after TBI has not yet been reported. Further research is needed to evaluate the potential value of FSP1 in targeting ferroptosis to treat TBI.

Transient receptor potential canonical channel 6 (TRPC6) is a nonselective cation channel protein that may lead to brain injury and brain diseases via nonselective transport of iron [25]. In a TBI mouse model, overexpression of TRPC6 was observed in astrocytes and neurons in the damaged area of brain tissue [25].

The voltage-dependent anion channel (VDAC) is a transmembrane channel that transports ions and metabolites [52]. Yagoda et al. [53] reported that erastin could act on VDACs. Following siRNA intervention to alter the expression of VDAC2 or VDAC3, they found that cells developed tolerance to ferroptosis caused by erastin. However, as overexpression of VDAC2 and VDAC3 did not improve cell sensitivity to ferroptosis caused by erastin, VDAC2 and VDAC3 are necessary but insufficient conditions for ferroptosis. Furthermore, methionine (Met) can be converted into cystine via a sulfur transfer pathway to synthesize GSH under oxidative stress, which helps GPXs inhibit the generation of lipid reactive oxygen and prevent oxidative cell damage. As a result, the sulfur transfer pathway can inhibit the occurrence of ferroptosis [54].

In summary, ferroptosis is activated and controlled by various induction or regulation pathways after TBI. Moreover, there are time differences in the peak expression of relevant indicators of ferroptosis after TBI, such that imbalance of the redox system occurs earlier and iron deposition and lipid peroxidation occur later. These findings confirm the occurrence of ferroptosis after TBI, both in the acute and chronic phases, and provide multiple targets for potential TBI treatment targeting ferroptosis. Interestingly, other cell modes of death can also regulate ferroptosis in addition to the aforementioned pathways. It was found that not only ferroptosis but also necroptosis, autophagy, apoptosis, and pyroptosis occurred after TBI and they interacted with each other. Studies have shown that the activation of ferroptosis is dependent on autophagy [55]. However, each cell mode of death needs to undergo a period of activation and may be present during both the acute and chronic phase after TBI; therefore, the timing of ferroptosis and other cell modes of death after TBI needs further research.

## 3. Ferroptosis and Other Cell Models of Death after TBI

**Figure 2:**
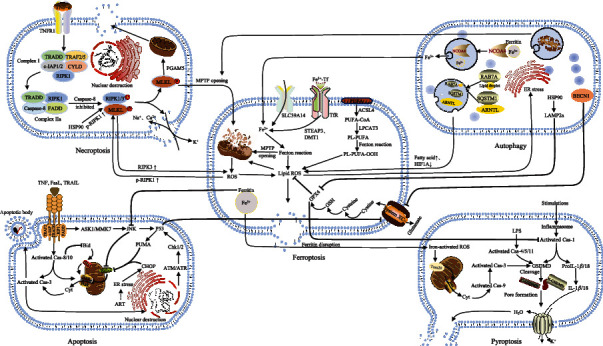
The neurological links and mutual regulatory mechanisms between ferroptosis and other models of cell death including necroptosis, autophagy, apoptosis, and pyroptosis. Top left shows that HSP90 and MPTP opening are the positive factors of both necroptosis and ferroptosis. Top right shows that several selective autophagy including ferritinophagy, lipophagy, clockophagy, BECN1-mediated system XC- inhibition, HSP90, and mitochondrial dysfunction promote ferroptosis by degrading ferritin, lipid droplets, ARNTL, and GPX4. Ferroptosis induces autophagy by promoting ER stress. Down left shows ferroptosis inhibits apoptosis through reducing the JNK signaling pathway activity. The p53 is the positive factors of both apoptosis and ferroptosis. Down right shows that ferroptosis promotes pyroptosis by improving iron-activated ROS through disrupting ferritin while suppresses pyroptosis by inhibiting Cas-1 activity with GPX4. TRADD: TNF receptor-associated death domain; TRAF: TNF receptor-associated factor; c-IAP: inhibitor of apoptosis protein; CYLD: deubiquitinase cylindromatosis; PGAM5: phosphoglycerate mutase family member 5; p-RIPK1: phosphorylated RIPK1; ARNTL: aryl hydrocarbon receptor nuclear translocator like; LAMP2a: lysosomal-associated membrane protein 2; FasL: ligand of fas cell surface death receptor; FADD: FAS-associated death domain; ASK1: apoptosis signal-regulating kinase; MMK7: mitogen-activated protein kinase kinase 7; Cas: caspase; Bid: proapoptotic Bcl-2 homology interacting-domain death agonist; tBid: truncated Bid; Bax: Bcl-2-associated X protein; Bak: Bcl-2 homologous antagonist killer; Cyt: cytochrome; PUMA: p53 upregulated modulator of apoptosis; CHOP: C/EBP-homologous protein; Chk: cell cycle checkpoint kinase; ATM: serine/threonine kinase; ATR: serine/threonine-protein kinase; ART: artesunate; ER: endoplasmic reticulum; LPS: lipopolysaccharide; IL: interleukin.

As previously described, TBI can lead to causes of neuronal cell death other than ferroptosis, including necroptosis, autophagy, apoptosis, and pyroptosis [6]. Oxidative stress, which inevitably occurs after TBI, generates a large amount of ROS that attack the phospholipid membrane rich of PUFAs to cause lipid peroxidative damage [10]. Lipid peroxidation is thus an important link in the different models of cell death following TBI. Ferroptosis differs from necroptosis, autophagy, apoptosis, and pyroptosis in morphological features. Ferroptosis is mainly manifested as small mitochondria with increased mitochondrial membrane densities, reduction or vanishing of mitochondria cristae, and rupture of outer mitochondrial membrane, while the nucleus is normal [56, 57]. Necroptosis results in rupture of plasma membrane, swelling of cytoplasm and organelles, moderate chromatin condensation, and spillage of cellular components [11]. Autophagy can form autophagosomes with double-membrane structure and vacuolize the cytoplasm, but no chromatin condensation [58, 59]. Apoptosis is characteristic by DNA fragmentation, destruction of the nuclear proteins and cytoskeleton, crosslinking of proteins, and the formation of apoptotic bodies [60]. Pyroptosis leads to cell swelling, pore formation, membrane rupture, and massive leakage of cytoplasmic components [61]. The links and mutual regulatory mechanisms between ferroptosis and other models of cell death are discussed below (Figure 2).

### 3.1. Ferroptosis and Necroptosis

Necroptosis is a programmed form of cell death that can regulate necrosis with passive and active proinflammatory functions [62]. Numerous reports have indicated that ferroptosis is always accompanied by necroptosis. Molecular markers of ferroptosis and necroptosis were increased following ICH *in vitro* and *in vivo*, and neuron ultrastructure after ICH was characterized by both ferroptosis and necroptosis [63, 64]. Necroptosis and ferroptosis also were observed in mouse hippocampus after CUMS and in a heat stress model in broilers [65, 66]. Activation of necroptosis is dependent on receptor-interacting protein kinase 1/3 (RIPK1/3) and mixed lineage kinase domain-like protein (MLKL) [67]. The summary steps are tumor necrosis factor (TNF) signalling and the deubiquitination of receptor-interacting protein (RIP) 1, phosphorylation of RIP1 and RIP3, caspase-8 inactivation, and the phosphorylation of MLKL [60]. In a mouse model of renal ischemia-reperfusion injury [68], cells with knockout of the ferroptosis-related factor ACSL4 using CRISPR/Cas9 gene editing showed time-dependent sensitivity to necroptosis, while cells with knockout of the necroptosis-related protein MLKL were more prone to ferroptosis. Cao et al. [65] revealed abnormal expression of necroptosis-related proteins such as RIPK3, p-MLKL, ferroptosis-related protein FTL, and lipid peroxidation using proteomic techniques in the hippocampus of chronic unpredictable mild stress model mice. As previously described, ferroptosis could cause mitochondrial damage, which led to mitochondrial permeability transition pore (MPTP) opening. Subsequently, RIP1/3 phosphorylation was further exacerbated and eventually led to necroptosis [69]. Interestingly, RIP3 upregulation excessed ROS production and induced MPTP opening via the endoplasmic reticulum stress (ER stress)/calcium overload/ROS pathway, which increased the sensitivity of cells to ferroptosis [69–74]. In addition, heat shock protein 90 (HSP90), cysteine, and NADPH also were believed as a link between ferroptosis and necroptosis [69, 75, 76]. These findings demonstrate that there is a crosstalk between ferroptosis and necroptosis, but the regulatory relationship between necroptosis and ferroptosis still requires further study.

### 3.2. Ferroptosis and Autophagy

Autophagy [77] refers to the process in which the detached double membrane of the ribosome-free attachment zone of the rough endoplasmic reticulum wraps cytoplasm, organelles, proteins, and other cellular components that need to be degraded to form autophagosomes. Next, the autophagosomes fuse with lysosomes to form autophagolytic lysosomes that degrade the enclosed contents to achieve the cell's metabolic needs and organelle renewal. Accumulating evidence indicates that ferroptosis requires the autophagy machinery for its execution and is a type of autophagy-dependent cell death [55, 78]. Prior studies have found that HSP90, an evolutionarily conserved molecular chaperone, plays a role in ferroptosis by regulating the expression of Lamp-2a in the autophagy pathway [79]. Autophagy was also observed to degrade ferritin in neurons to increase free-iron levels in a mouse model of subarachnoid hemorrhage, thereby promoting ferroptosis [80]. Recent studies have found that autophagy contributes to ferroptosis through degrading the ferroptosis-related protein, ferritin with the autophagy-related gene 5 (ATG5)-ATG7-nuclear receptor coactivator (NCOA4) autophagic pathway [81, 82]. Knockout of *Atg5* and *Atg7* limited erastin-induced ferroptosis with decreased intracellular Fe^2+^ levels and lipid peroxidation. Knockdown of *NCOA4* (a selective cargo receptor for the autophagic turnover of ferritin) by specific shRNA in PANC1 or HT-1080 cells increased FTH expression and inhibited erastin-induced death, while overexpression of *NCOA4* by gene transfection in PANC1 cells suppressed FTH expression and increased erastin-induced death [81, 82]. In addition, RAB7A-mediated lipophagy, BECN1-mediated system XC- inhibition, STAT3-mediated lysosomal cell death, and SQSTM1-mediated clockophagy also were shown to regulate ferroptosis [83]. Tian et al. [84] determined that FTH regulated ferroptosis via ferritinophagy in the 6-OHDA model of PD, which suggested there is a positive relationship between ferritinophagy and ferroptosis and FTH is a key link between these processes. These studies all reveal that autophagy can regulate ferroptosis, which provides a new hypothesis to treat TBI via targeting autophagic ferroptosis.

### 3.3. Ferroptosis and Apoptosis

Apoptosis is a classic mode of programmed cell death involving extracellular and intracellular pathways [85]. The extracellular pathway is activated by receptors on the cell membrane such as TNF-*α*, while the intracellular pathway is mainly affected by the permeability of the mitochondrial outer membrane and regulation of the Bcl-2 protein family. Some studies have reported an interrelationship between ferroptosis and apoptosis. Zheng et al. [86] found that apoptosis can be converted to ferroptosis. Studies of tumors have shown that the ferroptosis inducer erastin activates the p53-dependent CHOP/PUMA axis and increases sensitivity to apoptosis induced by the tumor necrosis factor-related apoptosis-inducing ligand (TRAIL) [87]. FTH has been shown to inhibit apoptosis through the JNK signaling pathway activity [88]. Thus, there are some crosstalks between ferroptosis and apoptosis.

### 3.4. Ferroptosis and Pyroptosis

Pyroptosis, which is also termed cell inflammatory necrosis, is a mode of programmed cell death dependent on inflammatory caspases (caspase-1 or caspase-11 in mice; caspase-1, caspase-4, or caspase-5 in human) [89]. TBI can cause tissue destruction and hemorrhage that lead to the release of damage-associated molecular patterns (DAMPs), which are recognized by the NOD-like receptor protein 3 (NLRP3) and promote inflammasome formation [90]. Inflammasomes may activate caspase-1 directly or indirectly. Activated caspase-1 promotes the formation of functional mature bodies by cleaving the IL-1*β* precursor and IL-18 precursor and induces the opening of cell membrane and pyroptosis by cleaving gasdermin D (GSDMD). Pyroptosis is characterized by nuclear pyknosis, cell swelling, the formation of lipid membrane vacuoles at the plasma membrane, and eventual rupture without DNA fragmentation [64]. Both pyroptosis and ferroptosis are accompanied by damage to the cytoplasmic membrane; in particular, ROS-mediated damage to the cytoplasmic membrane may involve reciprocal regulation between pyroptosis and ferroptosis. In addition, both two cell death forms were found to be induced by increased intracellular iron and ROS, which illustrates that iron manipulation and ROS elevation may be a common stimulus for both ferroptosis and pyroptosis [91]. The above study also shows that pyroptosis and ferroptosis are synergistic. Moreover, the ferritin disruption results in ROS elevation in an iron-dependent manner, and pyroptosis can be induced by iron-activated ROS via a Tom20-Bax-caspase-GSDME pathway [92]. Kang et al. [93] have found that the deficiency of GPX4 in myeloid cells increased the production of caspase-1/11-mediated GSDMD and promoted pyroptosis. These studies suggest crosstalks between ferroptosis and pyroptosis, but the mechanisms of which warrant further exploration.

The above evidence of the neurobiological links between ferroptosis and various modes of cell death is derived from animal models of other injuries or diseases, but not TBI. Although a direct reciprocal regulatory relationship between ferroptosis and other modes of cell death after TBI has not yet been reported, oxidative stress is involved in various forms of cell death and has been widely observed after TBI. Through further research on the mechanism of TBI, links between ferroptosis and other modes of cell death after TBI may be identified and investigated for multitarget, comprehensive treatment of TBI.

## 4. Ferroptosis-Based Treatment of TBI

TBI can cause neuronal damage and death and is often complicated by neurological dysfunctions including motor deficits, cognitive dysfunction, dementia, and mental illnesses such as anxiety and depression [6]. The aforementioned studies have demonstrated that iron deposition, lipid peroxidation, and GSH depletion are involved in neurodegeneration and neurological dysfunction after TBI. The following sections summarize the research to date targeting ferroptosis for the treatment of TBI (Figure 1 and Table 1).

### 4.1. Protective Effects of Ferroptosis Inhibitors against TBI

Liproxstatin-1 (Lip-1), which is also known as a reactive oxygen scavenger, is a potent ferroptosis inhibitor containing amide and sulfonamide subunits that can prevent ferroptosis caused by inhibition of xCT or/and inactivation of GPX4 by capturing ROS to directly inhibit lipid peroxidation [94]. A study has shown that Lip-1 can reduce heme-induced neuronal damage, neurological deficits, neuronal degeneration, cerebral edema, microglia activation, and neuritis by inhibiting ferroptosis (e.g., by promoting the expression of GPX4, inhibiting the expression of ACSL4, and reducing the generation of 5-hydroxyeicosatatraeniocacid) and protect the integrity of the blood-brain barrier (BBB) in an animal model of subarachnoid hemorrhage [12]. In our previous study [23], we noted that Lip-1 reduced brain lesion volume and neurodegeneration and improved cognitive dysfunction in a mouse TBI model. Furthermore, the neuroprotective effect of Lip-1 on TBI was found to relate to the reduction of iron levels and lipid peroxides like MDA and the restoration of GSH in the injured cortex. Lip-1 also regulated the expression levels of ferroptosis-related proteins (e.g., xCT, COX2, TrR1, Fpn, FTH, FTL, NOX2, and 4-HNE) and genes (*Slc7all*, *Ptgs2*, *tfr1*, *Fpn*, *Fth*, and *Ftl*) following TBI. The above results indicate that Lip-1 may play a neuroprotective role by inhibiting the ferroptosis pathway to improve the outcomes of TBI, suggesting a new direction for clinical treatment of TBI.

Ferrostatin-1 (Fer-1), a synthetic antioxidant containing arylalkylamine, is widely used as a ferroptosis inhibitor to prevent membrane lipid damage through a reduction mechanism. To test the effect of ferroptosis after TBI, researchers injected Fer-1 into the lateral ventricle. Notably, they found that Fer-1 reduced iron deposition, neurodegeneration, and brain lesion volume and improved long-term outcomes in terms of both motor and cognitive impairment following TBI [14]. However, as lateral ventricle injection is not clinically feasible, future studies could perform intraperitoneal and tail intravenous injection of Fer-1 and observe its therapeutic time window.

Ferristatin II (Fer-II), which is an iron uptake and TfR1 inhibitor, downregulates TfR1 via receptor degradation [95]. TfR1, a specific ferroptosis marker, is largely responsible for Tf-mediated delivery of iron [96, 97]. A previous study found that administration of Fer-II reduced the expression of TfR1 in neurons and thus played a neuroprotective role by alleviating the neuron damage and neurodegeneration caused by TBI [98]. The molecular mechanisms of Fer-II include reducing Fe^3+^ content and iron deposits; reversing the expression of iron homeostasis-related proteins like TfR1, lipid peroxidation genes, and protein; and reducing the high level of MDA after TBI [98].

Deferoxamine (DFO), which is an iron scavenger, can promote angiogenesis by upregulating vascular growth factors like HIF1-*α*, providing nutrition for neuron, and supporting cell migration and axon growth [99, 100]. After CNS injury, the accumulated ROS caused by ischemia and inflammation lead to oxidative injury [101, 102]. DFO can form a stable complex with free iron and inhibit the ROS formation induced by iron to repair oxidative damage [102, 103]. Prior research has shown that DFO can reduce iron, FTL, FTH, Tf, and TRPC6 levels in mice at 28 days after TBI and alleviate brain lesions and cognitive dysfunction in mouse TBI model [25].

### 4.2. Protective Effects of Antioxidants against TBI

Baicalein, a flavonoid with high content in *Scutellaria baicalensis*, is a polyphenolic antioxidant 12/15-LOX inhibitor with anti-inflammatory and antioxidant effects that is widely used in clinical practice [104]. *In vitro* studies have shown that the ferroptosis inducer RSL3 (GPX4-specific inhibitor) and mechanical stretching can induce the death of HT22 cells and that this can be inhibited by Fer-1, triacsin C (ACSL4 inhibitor), and baicalein [105]. Liquid chromatography-tandem mass spectrometry (LC-MS/MS) has been used to reveal increased AA/AdA-PE, increased expression of 15-LOX and ACSL4, and GSH depletion in the brain tissue of TBI mice. However, after baicalein injection, the aforementioned ferroptosis biomarkers were reversed, TUNEL-positive cells in the hippocampus decreased, and memory dysfunction improved after TBI, suggesting that its neuroprotective effect might also relate to suppression of 12/15-LOX [105]. The above findings shed light on the protective mechanism of baicalein in TBI-inhibiting ferroptosis and provide a theoretical basis for its clinical application in TBI.

Melatonin, also known as N-acetyl-5-methoxy-tryptamine, is a hormone secreted by the pineal gland with broad antioxidant properties [106]. Previous studies have demonstrated that melatonin can exert neuroprotective effects against TBI through antiapoptosis, antioxidation, and anti-inflammatory mechanisms [106]. Recent studies have shown that melatonin improves neurological dysfunction after TBI by reducing iron deposition and neurodegeneration [23]. Further studies have found that melatonin exerts a neuroprotective role by inhibiting ferroptosis, which is mainly dependent on the melatonin receptor 1B (MT2). However, melatonin did not exert a neuroprotective effect in a mouse TBI model with knockout of neuronal *Fth*. Through both *in vivo* and *in vitro* experiments, Wu et al. [107] demonstrated that melatonin significantly improved brain function in mice after TBI by attenuating ferroptosis and ERS and alleviating lipid peroxidation via circPtpn14/miR-351-5p/5-LOX signaling. Taken together, these findings suggest that melatonin is an effective ferroptosis inhibitor. Thus, the antiferroptosis mechanism of melatonin could be leveraged for TBI therapy.

Polydatin, which is a single crystal extract derived from the plant *Polygonum cuspidatum*, has strong antioxidant and neuroprotective effects [108]. Recent studies have demonstrated that polydatin can protect neurons and improve motor deficits in mice TBI models and reverse free-iron deposition, increase MDA, and decrease GPX4 activity in injured brain areas [109]. In addition, polydatin was shown to recover the expression levels of *Gpx4*, *Slc7a1*, *Ptgs2*, and *Atp5g3*. To further verify the antiferroptosis effect of polydatin, researchers cultured Neuro2A cells and administered hemin to induce injury. They found that the antiferroptosis effect of polydatin was better than that of Fer-1 and mainly involved inhibition of the GPX4 pathway. Overall, these studies suggest that polydatin is a potential drug targeting ferroptosis for the treatment of TBI.

### 4.3. Other Drugs

Ruxolitinib is a JAK1/2 inhibitor used for the treatment of myelofibroma. Studies have shown that ruxolitinib exerts neuroprotective effects by inhibiting the JAK-STAT pathway in an experimental TBI model [110]. Our research group previously reported [24] that ruxolitinib exerts protective effects by inhibiting iron deposition, neurodegeneration, brain edema, and brain lesion volume in a mouse TBI model, which were manifested by improved motor deficits, memory dysfunction, and anxiety-like behaviors. A further study found that ruxolitinib can reverse decreases in GPX4 and increases in TfR1, COX2, and 4-HNE during the acute phase after TBI. Moreover, the antiferroptosis effect of ruxolitinib was better than that of the ferroptosis-specific inhibitor Fer-1 in a mouse TBI model [24]. Ruxolitinib, as a clinical drug, is thus a pleiotropic neuroprotective agent with potential applications for the treatment of TBI patients.

MicroRNA (miRNA), a negative regulator molecule, has siRNA-like effects and inhibits mRNA translation or directly degrades mRNA by targeting proteins and/or genes [111]. TBI has been previously linked to miRNA abnormalities [112]. Specifically, it was reported that miR-212-5p was downexpressed in the extracellular vesicles of injured brain tissue after TBI [113]. Xiao et al. [34] found that low expression of miR-212-5p promoted ferroptosis in HT-22 and Neuro-2a neuronal cell lines, which was at least partly by targeting *prostaglandin-endoperoxide synthase-2* (*Ptgs2*). In addition, studies [34] have shown that miR-212 is highly expressed in the brain and plays an important role in synaptic plasticity, memory formation, and maintaining BBB integrity, while administration of miR-212-5p to TBI model mice significantly improves learning and spatial memory. Thus, miRNA is a potential treatment for TBI that can regulate ferroptosis and other mechanisms.

N-acetylcysteine (NAC) has a neuroprotective effect that is associated with activation of the Nrf2 antioxidant response element signaling pathway in TBI model mice. Nrf2 regulates xCT and GPX4, which inhibit the initiation of ferroptosis and promote the expression of target genes mediating cellular antioxidant and iron metabolism states [38, 114]. A controlled series of clinical trials found that NAC supplementation significantly alleviated the symptoms of mild TBI sequelae in the short term [115].

Pioglitazone is a specific peroxisome proliferator-activated receptor-*γ* (PPAR*γ*) agonist. PPAR*γ* is a nuclear transcription factor and most strongly expressed in the adipose tissue and the immune system [116]. PPAR*γ* forms a heterodimer with retinoid X receptor (RXR) and subsequently binds to DNA to regulate lipid metabolism and suppresses neuronal inflammation [116, 117]. Previous studies suggested that PPAR*γ* activation has a protective effect on the CNS injury [118–121]. Liang et al. [118] found that PPAR*γ* decreased in neurons subjected to ferroptosis after TBI, but pioglitazone can reverse it. Further study found that PPAR*γ* blocked neuronal ferroptosis [118]. Interestingly, pioglitazone administration can effectively reduce COX2 and MDA levels, in addition to attenuating the neurological severity score (NSS), the injured area, and neuronal loss of the mice with TBI. The above results indicate that pioglitazone has the potential antiferroptosis effects in treatment of TBI. Likewise, the PPAR*γ* agonist rosiglitazone (RSG) also has a similar effect with pioglitazone. RSG exerts neuroprotective effects via attenuating inflammation and CA3 neuronal loss and the suppression of neuronal autophagy and apoptosis in the cortex following TBI in rats, which also may be exploited to improve clinical treatment in TBI [122, 123].

Tetrandrine (Tet) is a natural bisbenzylisoquinoline alkaloid, which presents favorable anticancer activity, anti-inflammatory, and analgesic activity [124, 125]. Previous studies suggested Tet ameliorated cognitive dysfunction through suppressing microglial inflammatory activation and neurotoxicity in the 5XFAD mouse and attenuated ischemia/reperfusion- (I/R-) induced neuronal damage in the subacute phase by decreasing oxidative stress, apoptosis, and autophagy [126, 127]. A study [124] found that Tet treatment improved the modified NSS (mNSS) after TBI, reduced brain contusion lesions and cerebral edema of TBI mice, elevated the GPX4, GSH, SCL7A11, and FTH expressions, and reduced MDA levels in TBI mice. A further study found that Tet enhanced autophagy-related proteins, BECN1 and LC3II/I expressions, while reduced p62 expression, which demonstrated that Tet could ameliorate TBI by activating autophagy to reduce ferroptosis. These findings suggest that Tet may be a clinical drug to treat TBI.

In summary, inhibiting ferroptosis is a feasible therapeutic strategy to exert neuroprotective effects against TBI. Further understanding of ferroptosis will provide new insights for drug-targeted therapy and evaluation of TBI patients by identifying additional ferroptosis-related molecular regulatory mechanisms.

## 5. Conclusion and Future Directions

TBI is a leading cause of mortality in young adults and a major cause of death and disability across all ages in all countries, but there are currently no clinical guidelines or recommendations for treatment of TBI [2]. Ferroptosis is a form of regulated cell death caused by iron-dependent peroxidation of lipids [51]. In recent years, a series of secondary brain injuries caused by TBI—including ion channel disruption, excitatory neurotransmitters and iron release, oxidative stress, lipid peroxidation, ROS accumulation, and mitochondrial dysfunction—closely related to ferroptosis have been identified. Thus, this article reviewed the ferroptosis pathways, related regulatory mechanisms, and the role of ferroptosis in TBI with the aim of providing new insights for treating TBI.

In addition to animal researches, numerous studies have confirmed the complex pathophysiological relationship between ferroptosis and human brain injuries and diseases. The biomarkers of ferroptosis in amyotrophic lateral sclerosis (ALS) were associated with clinical functional decline by following 109 ALS patients [128]. A marked dysregulation of 4-HNE, GSH, and GPX4 by measuring ferroptosis biomarkers in blood from 83 unrelated children with a clinical diagnosis of epilepsy and 44 age-matched controls confirms a crucial role for ferroptosis in epilepsy [129]. Decreased Fpn expression and abnormal iron deposition significantly increased NOX4, 4-HNE, and MDA levels in damaged astrocytes of cerebral cortex, and five hub genes (*JUN*, *SLC2A1*, *TFRC*, *ALB*, and *NFE2L2*) closely related to ferroptosis were identified in Alzheimer's disease (AD) patients [130–132]. It was discovered by analyzing the human glioma genome that ferroptosis-related gene signature can be applied to low-grade glioma border localization and detection and predict glioma cell death and glioma patient progression [133–136]. DFO is effective in treating ischemic stroke in a randomized clinical trial [137]. These findings suggest that ferroptosis is a potential target for treatment for TBI.

Animal experiments have proved that various drugs, such as ferroptosis inhibitors and antioxidants, can reverse iron deposition and the expression of related molecules in the ferroptosis pathways to have a positive treatment effect for TBI. However, given the short research history of both ferroptosis and TBI, there is currently a limited study on ferroptosis after TBI, especially mentioning human patients, rodent, or large animal models. Meanwhile, the molecular mechanisms of ferroptosis post-TBI are not completely elucidated, and the treatment of TBI using ferroptosis inhibitors has not yet achieved satisfactory results in clinical trials. In addition, the existing studies have mainly focused on positive effects with no attention paid to potential side effects of the drugs. Therefore, TBI human samples can be used to verify the pathophysiological mechanism and clinical significance of ferroptosis, and bioinformatics analysis can be used to predict the role and value of ferroptosis in TBI to reveal the molecular mechanisms of ferroptosis and TBI in the future to provide novel targets for the treatment of TBI.

## Figures and Tables

**Table 1:** Effects of ferroptosis-related drugs on TBI and their potential mechanisms.

Drugs	Drug administration	Targets	Neuroprotective effects	Potential mechanisms	Article, year
Lip-1	10 mg/kg, once daily, i.p.	Activate xCT/GS H/GPX 4 pathway	Brain lesion volume↓, cytoplasmic shrinkage or nuclear pyknosis↓, neurodegeneration↓, improve cognitive dysfunction	Iron deposition↓, MDA↓, GSH↑, xCT↓, COX2↓, TfR1↓, Fpn↓, FTH↓, FTL↓, NOX2↓, 4-HNE↓, *Slc7a11*↓, *Ptgs2*↓, *Tfr1*↓, *Fpn*↓, *Fth*↓, *Ftl*↓	Rui et al., 2021 [23]
Fer-1	3 *μ*M, i.c.v.	Inhibit lipid peroxidation	Brain lesion volume↓, neurodegeneration↓, improve cognitive dysfunction	Iron deposition↓	Xie et al., 2019 [14]
Fer-II	10 mg/kg, twice daily, i.p.	Iron chelator and TfR1 inhibitor	Neuronal damage↓, neurodegeneration↓, cytoplasmic shrinkage↓, nuclear pyknosis↓	Iron deposition↓, MDA↓, TfR1↓, Tf↓, Fth↑, COX2↓, *Gpx4*↑, *SOD1*↓	Cheng et al., 2022 [98]
DFO	100 mg/kg, once daily, i.p.	Iron chelator	Brain lesion volume↓, improve cognitive dysfunction	Iron deposition↓, FTH↓, FTL↓, Tf↓, TRPC6↓	Zhang et al., 2013 [25]
Baicalein	50 mg/kg, i.p.	Inhibit lipid peroxidation (12/15-LOX inhibitor)	Improve spatial memory acquisition	Hippocampal neuronal apoptosis↓, AA/AdA-PE↓, 15-LOX↓, ACSL4↓, GSH↑	Kenny et al., 2019 [105]
Melatonin	10 mg/kg, once daily, i.p.	Activate MT2 and inhibit FTH	Brain lesion volume↓, cytoplasmic shrinkage or nuclear pyknosis↓, neurodegeneration↓, improve cognitive dysfunction, alleviate anxiety-like behavior	Iron deposition↓, xCT↓, COX2↓, TfR1↓, Fpn↓, Nox2↓, Fth↓, Ftl↓, 4HNE↓, GSH↑, MDA↓, MT2↑, FTH↓, *Slc7a11*↓, *Ptgs2*↓, *Tfr1*↓, *Fpn*↓, *Fth*↓, *Ftl*↓, ERS↓, regulate circPtpn14/miR-351-5p/5-LOX signaling	Rui et al., 2021 [23] and Wu et al., 2022 [107]
Polydatin	50 mg/kg, i.p.	Activate GPX4 pathway	Acute neuronal damage↓, improve motor deficits and memory dysfunctions	Iron deposition↓, MDA↓, GPX4 activity↑, *Gpx4*↓, *Slc7a11↓*, *Ptgs2↓*, *Atp5g3*↓	Huang et al., 2021 [109]
Ruxolitinib	0.44 mg/kg, i.p.	Activate GPX4 pathway and inhibit TfR	Neurodegeneration↓, brain edema↓, brain lesion volume↓, improve motor deficits and memory dysfunctions, and anxiety-like behaviors, the shrinkage and hyperchromatic morphology↓	Iron deposition↓, GPX4↑, TfR1↓, COX2↓, 4-HNE↓	Chen et al., 2021 [24]
miR-212-5p agonist	5 nM, i.c.v.	Inhibit *Ptgs2*	Improve learning and spatial memory	miR-212-5p↑, *Ptgs2*↓, and ferroptosis↓	Xiao et al., 2019 [34]
Pioglitazone	5 mg/kg, i.p.	Activate PPAR*γ*	NSS↓, injured area↓, neuronal loss↓	PPAR*γ*↑, COX2↓, MDA↓	Liang et al., 2022 [118]
Tetrandrine	30, 45 or 60 mg/kg, once daily, i.p.	Regulate autophagy	Improve neurological function, cerebral edema↓, mNSS↓, brain contusion lesions↓	GSH↑, MDA↓, GPX4↑, BECN1↑, LC3II/I↑, p62↓, SCL7A11↑, FTH↑	Liu et al., 2022 [124]

Note. ↓: decrease; ↑: increase; i.p.: intraperitoneal; i.c.v.: intracerebroventricular.

## Data Availability

The data used or analysed during the current study are available from the corresponding author on reasonable request.

## Abbreviations

TBI: Traumatic brain injury
GSH: Glutathione
ERS: Endoplasmic reticulum stress
ROS: Reactive oxygen species
CNS: Central nervous system
Tf: Transferrin
TfR: Transferrin receptor
STEAP3: Six-transmembrane epithelial antigen of prostate 3
Fpn: Ferroportin
PCBP1/2: Poly C-binding protein 1/2
FTL: 24-heteromultimers of light chains
FTH: 24-heteromultimers of heavy chains
HO-1: Heme oxygenase-1
4-HNE: 4-Hydroxynonenal
GCS: Glasgow coma scale
ICU: Intensive care unit
PUFAs: Polyunsaturated fatty acids
AA: Arachidonic acid
AdA: Adrenic acid
ACSL4: Acyl-CoA synthetase long-chain family member 4
AA/AdA-PE: AA/AdA-phosphatidylethanolamine
LPCAT3: Lysophosphatidylcholine acyl transferase 3
AA/AdA-OOH-PE: AA/AdA-hydroperoxide-PE
LOX: Lipoxygenase
MDA: Malondialdehyde
COX2: Cyclooxygenase-2
NOX2: Nicotinamide adenine dinucleotide phosphate oxidases 2
*γ*-GCS: *γ*-Glutamate-cysteine synthetase
GS: Glutathione synthetase
GPX4: Glutathione peroxidase 4
GSSG: Oxidized glutathione
L-OOH: Lipid peroxide
TrxR1: Thioredoxin reductase 1
MVA: Mevalonate
EAAC1: Excitatory amino acid carrier type 1
NAD^+^: Nicotinamide adenine dinucleotide
SIRT2: Sirtuin 2
DPP4: Dipeptidyl-peptidase 4
FSP1: Ferroptosis-suppressor-protein 1
CoQ10: Coenzyme Q10
TRPC6: Transient receptor potential canonical channel 6
VDAC: Voltage-dependent anion channel
Met: Methionine
RIPK1/3: Receptor-interacting protein kinase 1/3
MLKL: Mixed lineage kinase domain-like protein
HSP90: Heat shock protein 90
TRAIL: Tumor necrosis factor-related apoptosis-inducing ligand
DAMPs: Damage associated molecular patterns
NLRP3: NOD-like receptor protein 3
GSDMD: Gasdermin D
Lip-1: Liproxstatin-1
BBB: Blood-brain barrier
Fer-1: Ferrostatin-1
Fer-II: Ferristatin II
DFO: Deferoxamine
LC-MS/MS: Liquid chromatography-tandem mass spectrometry
MT2: Melatonin receptor 1B
miRNA: MicroRNA
NAC: N-acetylcysteine
PPAR*γ*: Proliferator-activated receptor-*γ*.
